# The use of biological amniotic membranes in the treatment of recurrent macular holes

**DOI:** 10.1038/s41598-022-21754-8

**Published:** 2022-11-04

**Authors:** Gang Qiao, Lijun Xie, Qiangxing Zou, Chunmei He, Xiaojuan Zhang, Ziyan Tang, Shuai Zou, Kui Cao

**Affiliations:** Mianyang Wanjiang Eye Hospital, Mianyang, 621000 Sichuan China

**Keywords:** Diseases, Medical research

## Abstract

To evaluate the clinical therapeutic effects of a technique in which biological amniotic membranes (bAMs) are used in the treatment of patients with recurrent macular holes. In this prospective nonrandomized case series study, 23 eyes of 23 patients with recurrent macular holes who had already undergone surgery with pars plana vitrectomy with internal limiting membrane peeling were evaluated. In the surgery, a bAM was used to cover the macular area, and C_3_F_8_ tamponade was performed on these patients. Phacoemulsification combined with intraocular lens implantation was performed simultaneously in patients who had cataracts. Patients were followed up for at least half a year. The main outcomes were whether the macular hole closed, the morphological changes in the macular graft, the best-corrected visual acuity, intraocular pressure (IOP) and other indicators. In all eyes, the recurrent macular holes were closed. Two cases (8.69%, 2/23) had bAM shifting half a month after surgery, and these patients underwent a second surgery to adjust the position of the bAM and perform C3F8 tamponade. In the 6-month follow-up, 21 patients (91.30%, 21/23) had improved visual acuity (VA), and 2 patients (8.69%, 2/23) had no change in VA. The mean VA increased from 1.73 ± 0.32 before surgery to 1.12 ± 0.42 after surgery (t = 10.63, *P* = 0.00 < 0.01), and the mean IOP decreased from 22.13 ± 5.56 before surgery to 17.23 ± 1.71 after surgery (t = 5.14, *P* = 0.00 < 0.01). No serious complications occurred in any of the cases. The technique of using a biological amniotic membrane can be an effective treatment for patients with recurrent macular holes.

## Introduction

In the era of minimally invasive vitrectomy, the efficacy of macular hole surgery has greatly improved. However, there are still patients with macular holes that do not close or holes that reoccur^1^. Common causes of holes that do not close or holes that reoccur include large macular holes, pathological myopia combined with choroidal atrophy of macular holes, and macular holes due to trauma, among others^2^.

Patients with recurrent macular holes have often undergone pars plana vitrectomy (PPV) more than once. Therefore, the macular area in these patients may not have an adequate inner limiting membrane^3–5^. This means that the reoperations may be extremely difficult, and the results may be poor. In view of the above problems, we creatively used biological amniotic membranes (bAMs) to cover recurrent macular holes, and we achieved good results, which we report here.

## Materials and methods

In this prospective noncontrol case study, the cases were inpatients from Mianyang Wanjiang Eye Hospital. All participants signed informed consent forms. The experimental protocol was approved by the Medical Ethics Committee of Mianyang Wanjiang Eye Hospital and met the ethical requirements of clinical trials and the principles of the Declaration of Helsinki.

The inclusion criteria were as follows: (1) one or more PPV surgeries with ILM peeling and silicone oil (SO) tamponade were performed in our hospital or other hospitals in the past; (2) MH reopening was the main cause of recurrent retinal detachment; and (3) MH nonclosure, with the RD not being reattached for more than half a year.

The following exclusion criteria were applied: the patients could not undergo a second surgery, or a second surgery would provide little value because of systemic disease, ocular inflammation or blindness.

Preoperative examination: All participants were asked about their medical history and underwent a full ophthalmological examination before surgery. The examinations included visual acuity (VA), intraocular pressure (IOP), slit lamp microscopy, and fundus examinations; optical coherence tomography (OCT, Heidelberg Spectralis OCT) examinations of the macular area; and fundus photography (Opelpanoramic SLO, UK), among others. The best corrected visual acuity (BCVA) was converted to logMAR VA to facilitate statistical analysis.

Surgical techniques: Silicone oil (SO) was extracted during surgery in patients with silicone oil tamponade. Phacoemulsification (Phaco) combined with intraocular lens (IOL) implantation was performed simultaneously in patients with cataracts. All surgeries were performed by the same experienced surgeon (Gang Qiao) with 25-gauge PPV, a bAM for covering the MH and C3F8 tamponade. The specific methods were as follows. All patients underwent 3-port, 25-gauge PPV (Alcon, US). Then, the vitreous cavity was injected with triamcinolone acetonide (TA) to observe whether there was any residual vitreous cortex; if so, the residual vitreous cortex was thoroughly removed. Then, indocyanine green was used to stain the ILM to observe whether there was any residual ILM around the MH and whether the peeling range of the ILM was sufficient in the first surgery. The peeling range of the ILM was extended if necessary in the second surgery. Once the first two steps had been completed, the liquid under the retina was removed by fluid-air exchange. Peripheral retinal photocoagulation was performed if there were still holes or denatured areas in the peripheral retina. A 3 mm × 3 mm bAM transplant that had been presoaked in balanced saline was clamped with ILM tweezers, passed into the vitreous cavity through a 25-gauge valved trocar, and placed over the surface of the MH. The smooth epithelium side of the bAM transplant was placed face up. The transplant was adjusted to the middle of the MH so that the edge of the transplant extended past the edge of the MH. A flute needle was used to remove residual liquid around the transplant, which allowed the transplant to attach tightly to the retina and no longer move. Finally, 13% C3F8 gas was injected into the vitreous cavity, and the incision was sutured. After the surgery, the patients kept their face down or remained in a horizontal position to rest for 2 to 3 weeks to ensure that the MH closed. All patients were given anti-inflammatory and symptomatic treatment. At same time, we monitored VA, IOP, and postoperative complications (vitreous hemorrhage, hole reopening, endophthalmitis and postoperative intraocular hypertension) in these patients. All patients were reexamined by OCT and scanning laser ophthalmoscopy (SLO) at 2 weeks, 1 month, 3 months, and 6 months after surgery.

### Statistical analysis

SPSS 23.0 software was used for statistical analysis. The measurement data are presented as the mean ± standard deviation. An independent-sample *t* test was used for comparisons between groups, and differences for which *P* values were < 0.05 were considered statistically significant. A matched-sample *t* test was used for comparisons among groups, and differences for which *P* values were < 0.05 were considered statistically significant. The X^2^ test was used to compare count data between two groups. If 1 ≤ theoretical frequency < 5, chi-square was corrected. If the theoretical frequency was < 1, the exact probability method was used.

### Ethics approval and consent to participate

This study adhered to the tenets of the Declaration of Helsinki and was approved by the Ethics Committee of Mianyang Wanjiang Eye Hospital, Sichuan, China (WJYK-01). All participants provided informed consent.

## Results

A total of 23 eyes from 23 participants met the inclusion criteria, and all participants completed the treatment plan and a half-year follow-up. The participants were 12 males and 11 females with an average age of 60.83 ± 6.17 years (range 48 to 70 years). The mean preoperative BCVA was 1.71 ± 0.28 (logMAR), the average axis length (AL) was 27.67 ± 0.85 mm, the average intraocular pressure (IOP) was 22.13 ± 5.56 mmHg, and the mean preoperative MH size was 769.61 ± 257.02 μm. Among all eyes, 7 eyes had posterior scleral staphyloma, 5 eyes had traumatic macular holes, 9 eyes had high IOP secondary to silicone oil emulsification, 6 eyes had an IOL, and 13 eyes had cataracts. The basic clinical features before surgery are shown in Table 1.

**Table 1:** Patient characteristics.

Patient	Sex/age (years)	Eye	Axial length (mm)	MH (μm)	Posterior scleral staphyloma	Surgery (times)	Pre-BCV A (logMAR)	Post-BCVA (logMAR)	Pre-IOP (mmHg)	Post-IOP (mmHg)	MH status
1	F/57	L	27.2	732	NO	2	1.6	1.1	30	20.9	Closed
2	F/61	L	28.1	636	NO	3	1.5	1.0	17.3	16	Closed
3	F/66	L	29.3	1561	YES	3	1.7	1.7	18.9	17.2	Closed
4	M/65	R	27	1489	NO	2	2.5	2.5	16.5	16.2	Closed
5	M/54	R	28.1	634	NO	2	1.4	0.6	28.1	17	Closed
6	F/70	L	27	567	NO	3	1.7	1.3	27.6	18	Closed
7	F/69	R	28.1	598	NO	2	2	1.2	27.8	17.1	Closed
8	M/56	L	26.9	672	NO	3	1.2	0.7	19.6	16.5	Closed
9	F/59	R	28.3	702	YES	2	1.7	1.3	15	17.2	Closed
10	M/61	L	27	498	NO	3	1.7	1.1	14.7	14.8	Closed
11	F/63	R	28.3	477	YES	4	2	1.2	17.2	16.3	Closed
12	F/65	L	28.4	872	YES	2	2.5	1.6	29.1	19.3	Closed
13	M/59	L	28.6	912	YES	2	1.6	0.8	28.4	17	Closed
14	M/54	L	27.8	813	NO	2	1.6	0.9	20.1	19	Closed
15	F/48	R	27.5	752	NO	3	1.4	1.0	21	16.2	Closed
16	M/49	R	27.5	783	NO	2	1.5	0.6	19	18.2	Closed
17	F/61	R	26.8	691	NO	3	2	0.9	17.8	16.3	Closed
18	F/63	L	28.7	761	YES	2	1.6	0.9	18.3	16.2	Closed
19	M/55	R	28.2	594	NO	3	1.7	0.8	25.8	19	Closed
20	M/70	L	26.4	690	NO	2	2	1.4	29.4	17.4	Closed
21	F/67	L	29.1	677	YES	2	1.6	0.8	16.7	15	Closed
22	M/68	L	26.9	856	NO	2	1.5	1.1	19.6	14.6	Closed
23	M/59	R	27.1	734	NO	2	1.7	1.2	31	21	Closed

BCVA, best-corrected visual acuity; F = female; L = left; logMAR = the logarithm of the minimal angle of resolution; M = male; MH = macular hole; R = right.

**Figure 1:**
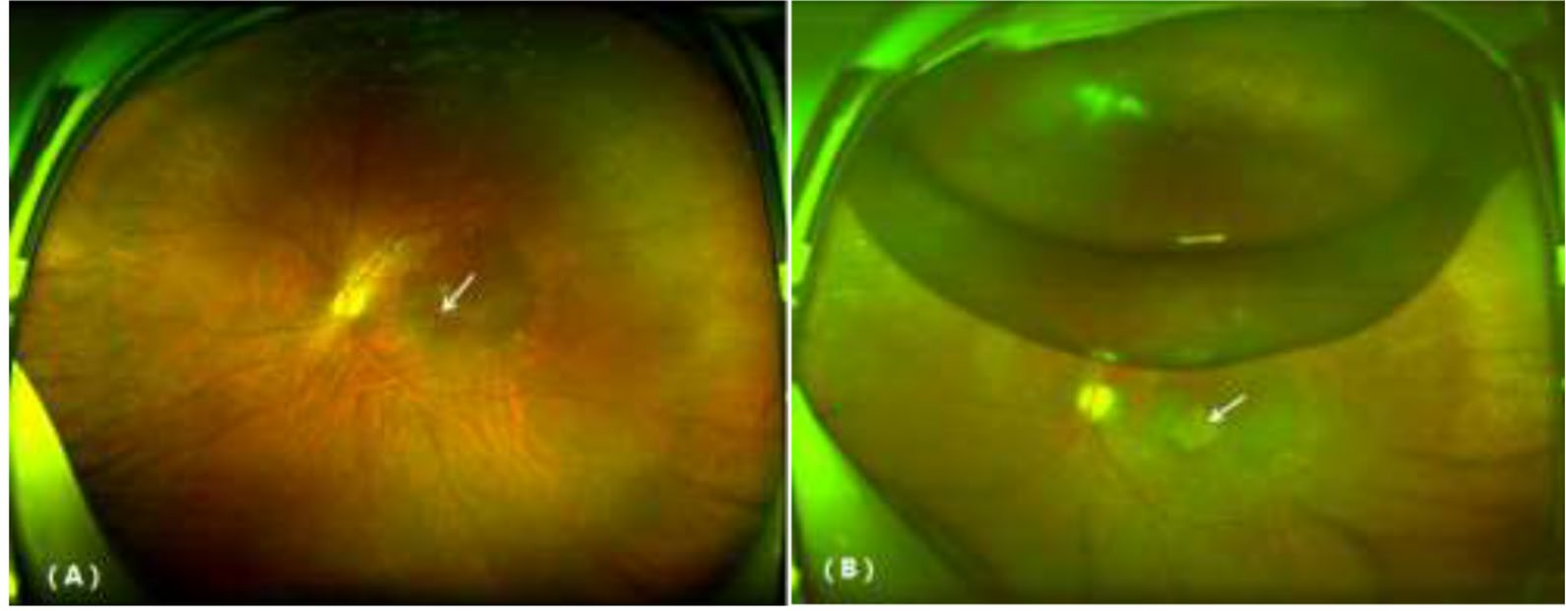
Comparison of SLO before and after treatment for a recurrent macular hole. (**A**) MH before surgery. (**B**) Two weeks after surgery, the MH was covered by the bAM (white arrow) and residual C3F8 gas in the vitreous cavity.

**Figure 2:**
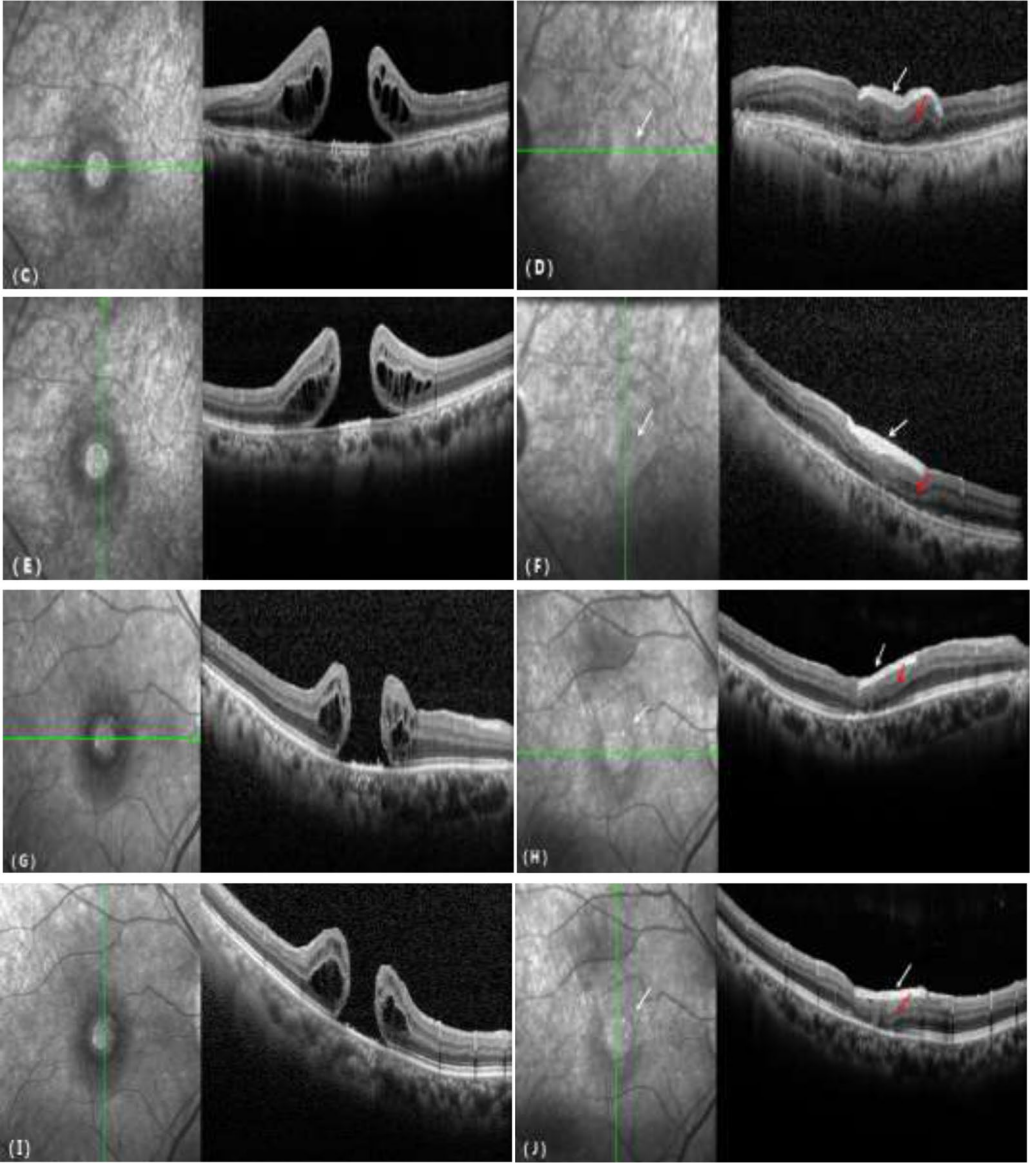
Comparison of OCT before and after treatment for a recurrent macular hole. (**C**, **E**, **G**, **I**) MH before surgery. (**D**, **F**, **H**, **J**) The MH was covered by the bAM (white arrow), the MH was closed, the outer structure of the retina was progressively repaired, and the ellipsoid zone was continuous (red arrow).

All patients successfully completed surgery, 9 eyes had silicone oil removed, and 13 eyes had cataract surgery performed simultaneously. At the final follow-up, the MH of all patients was closed, the retina was reattached, and there was no exposure of the RPE. The recovery conditions of typical cases are shown in Figs. 1 and 2. Two patients (8.69%, 2/23) were found to have a bAM translocation half a month after surgery and underwent a secondary surgery to adjust the bAM and perform C_3_F_8_ tamponade. After surgery, twenty-one patients (91.30%, 21/23) had improved best corrected visual acuity (BCVA), and two patients (8.69%, 2/23) had no change in BCVA. The mean BCVA increased from 1.73 ± 0.32 before surgery to 1.12 ± 0.42 after surgery (t = 10.63, *P* = 0.00 < 0.01), and the mean IOP decreased from 22.13 ± 5.56 before surgery to 17.23 ± 1.71 after surgery (t = 5.14, *P* = 0.00 < 0.01). A comparison of the indicators of the patients before and after surgery is shown in Table 2. No serious complications occurred in any of the cases.

**Table 2:** Comparison of the indicators of the patients before and a half-year after surgery.

Before or after surgery	Macular hole healing (case)	IOL	Axial length (mm)	BCVA (logMAR)	IOP (mmHg)
Before	0	6	27.67 ± 0.85	1.73 ± 0.32	22.13 ± 5.56
After	23	19	27.63 ± 0.81	1.12 ± 0.42	17.23 ± 1.71
*t*(χ^2^)/P value	*P* = 0.00	*P* = 0.00	t = 1.99 *P* = 0.06	t = 10.63 *P* = 0.00	*t* = 5.14 *P* = 0.00

## Discussion

The standard procedures for macular hole surgery include PPV, internal limiting membrane (ILM) peeling and intraocular tamponade. The cure rate of patients with idiopathic macular holes treated by these methods is more than 90%^1^. However, for special types of macular holes, such as pathological myopia-combined macular holes and recurrent macular holes, the cure rate can be as low as 60–70%^3^. For some patients with various causes of recurring macular holes, there is no residual ILM in their macular region to be peeled, and the use of some techniques (including ILM packing and the inverted ILM flap technique) is no longer effective. Therefore, the treatment of these diseases has become a difficult problem.

Some scholars have studied autologous or allogenic lens capsular flap transplantation^4^, autologous neurosensory retinal free flap (ANRFF) transplantation^5^ and other techniques for use in patients with recurrent macular holes. However, the above techniques are still associated with certain trauma and technical difficulty, and "the losses outweigh the gains". In particular, ANRFF requires cutting off a piece of peripheral retinal tissue, which may result in secondary harm to patients and cause complications such as choroidal and retinal hemorrhage. In addition, allogenic lens capsular flap and ANRFF transplantation require great surgical skill and therefore are not suitable for large-scale application.

In the past 2 years, amniotic membrane plugs have received more attention and favor because of their low cost and high MH healing rate. The so-called amniotic membrane technique uses a human amniotic membrane (hAM) as a kind of embolus, and an hAM is transplanted into the subretinal space between the retinal neuroepithelium and pigment epithelium, plugging the macular hole and promoting closure^6^. In fact, the use of an amniotic membrane as an alternative to conventional therapy is not a new concept, as it has been applied in various clinical specialties and plays a good role in promoting the treatment of many diseases. For example, bioamniotic membranes are used to assist in the treatment of otitis media^7^ with tympanic membrane perforation as well as in ocular surface reconstruction in surgery for severe ocular surface diseases^8^^.^ However, the use of amniotic membranes in the treatment of MH is innovative. The technique was used by Professor Tomaso Caporossi's team, who have published research results from more than 10 patients in the last 2 years ^9–12^; these results verified the role of amniotic membrane plugs in the treatment of patients with MH and retinal detachment with MH due to high myopia and the good clinical effects.

However, amniotic membrane plugs still need to be discussed, and their clinical applications need to be improved. The shortcomings of this technique can be summarized as follows: First, the hAM must be inserted between the RNL and the RPE during the surgery; therefore, the RPE under the MH may be touched or damaged, resulting in visual dysfunction. Second, because the hAM is inserted into the subretinal space, with the chorion layer facing the RPE, the structure of the macular area is disordered after anatomical closure, and the influence of macular function cannot be ignored. Finally, both hands of the surgeon are required during the surgery, and another operative incision must be made when a ceiling lamp is used. After the hAM plug is centered in the MH, a flute needle is inserted to drain the subretinal fluid beyond the edge of the posterior staphyloma, where a retinotomy is performed to ensure subretinal fluid evacuation. This two-step process increases the trauma caused by the surgery. Compared with ANRFF transplantation, hAM packing significantly reduces iatrogenic injuries and is associated with improved clinical outcomes, but based on the previous analysis, there is room for further improvement.

Based on previous studies, the project team of the author made improvements and proposed the bioamniotic membrane covering technique. The difference between this technique and that of hAM packing is that the bioamniotic membrane covers only the surface of the MH and does not damage the RPE below the MH. During healing, the MH still closes at its own anatomical level, and closure has no effect on the outer structure of the retina, which theoretically maximally protects the visual function of patients. During the surgery, the subretinal fluid is removed through the MH, and there is no need to add an extra retinal hole for internal drainage, which reduces the risk of iatrogenic injury. The surgery is simple and can be performed by the surgeon with only one hand, without a ceiling lamp or a fourth incision. As shown in Fig. 2, the bioamniotic membrane is contiguous with the macula surface, the macular structure is clear, the MH is completely closed, the outer structure of the retina progressively heals, and the ellipsoid zone is continuous.

The material used in this study was a bioamniotic membrane rather than a fresh amniotic membrane, as mentioned in the literature. The advantage is that the product is readily available and can be used directly, while fresh amniotic membranes require temporary processing of grafts, and there is a potential risk of infectious disease transmission^13^. The main disadvantage of the use of a bioamniotic membrane is that it provides fewer growth-promoting factors than a fresh amniotic membrane in the process of macular hole healing; however, according to our experimental results, there is no difference in the final therapeutic effect.

In summary, the bioamniotic membrane covering technique uses materials that are easy to obtain in the clinic and has potential advantages such as easy access, no damage and a short learning curve. The design of this technique avoids the disadvantages of previous surgical methods, improves the success rate of surgery for the treatment of recurrent macular holes, and theoretically maximizes the protection of patients' visual function. Because this study included only a small number of cases, a larger randomized controlled study with a longer follow-up period is needed to corroborate our results.

## Data Availability

The data used to support the findings of this study are available from the corresponding author upon request.
